# Supplementary material for: The emergence of macroscopic currents in photoconductive sampling of optical fields
Source: Nat Commun. 2022 Feb 18;13:962. doi: 10.1038/s41467-022-28412-7 (PMC8857260; doi:10.1038/s41467-022-28412-7)
Supplement: Supplementary file 1 — Supplementary Information [file 41467_2022_28412_MOESM1_ESM.pdf]

Supplementary Information: The emergence of macroscopic currents in photoconductive sampling of optical fields

Johannes Schötz et al.

SUPPLEMENTARY NOTE 1: PLASMA OSCILLATIONS

In the time-resolved induced charge signal on the electrodes, observed directly after the excitation, small oscillations are visible (cf. Figure 7a) of the main text). A magnified view is shown in Figure 1a). After the initial laser excitation at t=0, the oscillations are superimposed on the quick rise of the induced charge (black solid line) and they decay after some time. Both, the frequency of the oscillations as well as their decay depend on the gas pressure and the initial charge density created by the laser pulse.

We use the following simple approach to analyze the oscillations. The induced charge signal is Fourier-transformed and analyzed in the frequency domain, as illustrated in Figure 1b). In the spectrum (black solid line), a distinct peak is observed, which can be identified with the frequency f_{osc} of the superimposed oscillation. To cross-check this approach, an inverse Fourier-transform is performed on the spectrum multiplied with a Gaussian-filter around the oscillation frequency (red solid line). In the time domain (Figure 1a), good agreement between the extracted oscillations and the induced charge signal is observed. At higher pressures, the oscillations are quickly damped, which leads to a broader peak in the frequency domain and thus an increased uncertainty in determining the oscillation frequency.

We have extracted the oscillation frequency for series of simulations with varying pressures and intensities between 0.5 and $0.9{\cdot}10^{14}\,\mathrm{W\,cm^{-2}}$ (other parameters: $\omega_0 = 8\mu$m, $D = 100\,\mu$m, $\lambda_0 = 750$ nm and $T_{\mathrm{FWHM}} = 4.5$ fs), shown in Figure 1c). The scaling of the oscillation frequency is found to be close to half the plasma frequency f_{pl} evaluated from the peak plasma density n_{max} found at the center of the laser focus (solid lines):

$$f_{\mathrm{osc}} \approx 0.5 \cdot f_{\mathrm{pl}} = 0.5 \cdot \sqrt{\frac{n_{\mathrm{max}} e^2}{m_e \epsilon_0}} / 2\pi, \tag{1}$$

where e is the electron charge, m_{e} is the free electron mass and ϵ_0 is the vacuum permittivity. The additional proportionality factor of roughly 0.5 as well as the non-ideal scaling can be explained by the inhomogeneous charge distribution, since the charge density quickly decreases away from the center of the laser focus leading to a smaller effective charge density. In conclusion, the close relation of the extracted oscillation frequency to the plasma frequency shows that the observed oscillations can be identified as plasma oscillations.

SUPPLEMENTARY NOTE 2: WAVELENGTH AND KINETIC ENERGY SCALING

We performed simulations in order to investigate the scaling of the induced electrode charge with the wavelength of the driving laser. For this purpose, we changed the driving wavelength between 300 nm and 2000 nm while keeping the duration of the 1.75-cycle pulses constant in terms of number of cycles. The other parameters are close to the ones used in the simulations shown in the main text ($I = 8 \cdot 10^{13}\,\mathrm{W\,cm^{-2}}$, $\omega_0 = 8\,\mu$m, and $D = 85\,\mu$m).

The pressure-dependence of the induced charge on the electrodes at the end of the simulation for different wavelengths for nitrogen and argon is shown in Figure 2a) and c), respectively. The data is normalized to the maximum signal in nitrogen at 750 nm. In general, the signal amplitude grows as the wavelength increases. This can qualitatively be understood from the linear increase of the generated charge in the employed tunneling emission model (saturation can be neglected), since the temporal duration of the pulses scales linearly with wavelength. At low pressures, in the current regime, where charge interaction and electron atom scattering can be neglected, the wavelength scaling of the signal is strictly linear. This is illustrated for nitrogen and argon at 1 mbar (black dots and dashed line) in Figure 2b) and d), respectively.

At higher pressures, the electron-atom scattering as well as charge interaction start to play a role. For a fixed laser intensity, the average kinetic energy of the electrons scales with λ^2. This has two effects. Firstly, the electrons can overcome the restoring forces due to charge interaction more easily, which is discussed in the next section. Secondly, the energy dependence of the electron-atom scattering (cf. Figure 3a) of the main text) is reflected in the wavelength scaling of the signal. Since the mean-free path for low energy electrons (< 1 eV) is much higher in argon than in nitrogen, the induced charge for argon is larger at shorter wavelengths. However, as the mean-free path rapidly drops for argon at higher energies (> 1 eV), whereas it stays nearly constant in nitrogen, the signal increase in argon is

considerably smaller than in nitrogen. This behavior is also illustrated in the wavelength scaling of the induced charge at 50 mbar (red dots and dashed line) shown in Figure 2b) and d) for nitrogen and argon, respectively.

As discussed above, both the increase of the number of charges as well as the higher average kinetic energy of the electrons, which allow to more easily overcome restoring forces from the charge interaction, contribute to the signal growth with wavelength. In order to investigate the latter effect, we performed another set of simulations for nitrogen, where we only scaled the initial kinetic energy of the electrons while keeping all other laser parameters constant (cf. caption of Figure 3). The pressure dependence for different scaling parameter values is shown in Figure 3a). As the scaling parameter increases, the maximum of the induced charge signal grows and the pressure at which the maximum is obtained shifts to higher values. At the lowest pressures, in the current regime, however, the signal is practically constant, as shown by black dots in Figure 3b). This is expected, since in the current regime, electron scattering and charge interaction can be neglected.

The observed scaling of the maximum signal demonstrates that the charge interaction can partially be overcome with higher electron energies, as expected. Therefore, at a fixed ionization level, longer wavelength pulses can be beneficial since the average kinetic energy roughly scales with λ^2. Since we only consider direct electrons in our simulations, it would be interesting to investigate also the signal contribution of rescattering electrons that possess a higher average kinetic energy. We note, however, that the yield of rescattering electrons is typically many orders below the direct electrons and strongly decreasing with increasing wavelengths.

SUPPLEMENTARY FIGURES

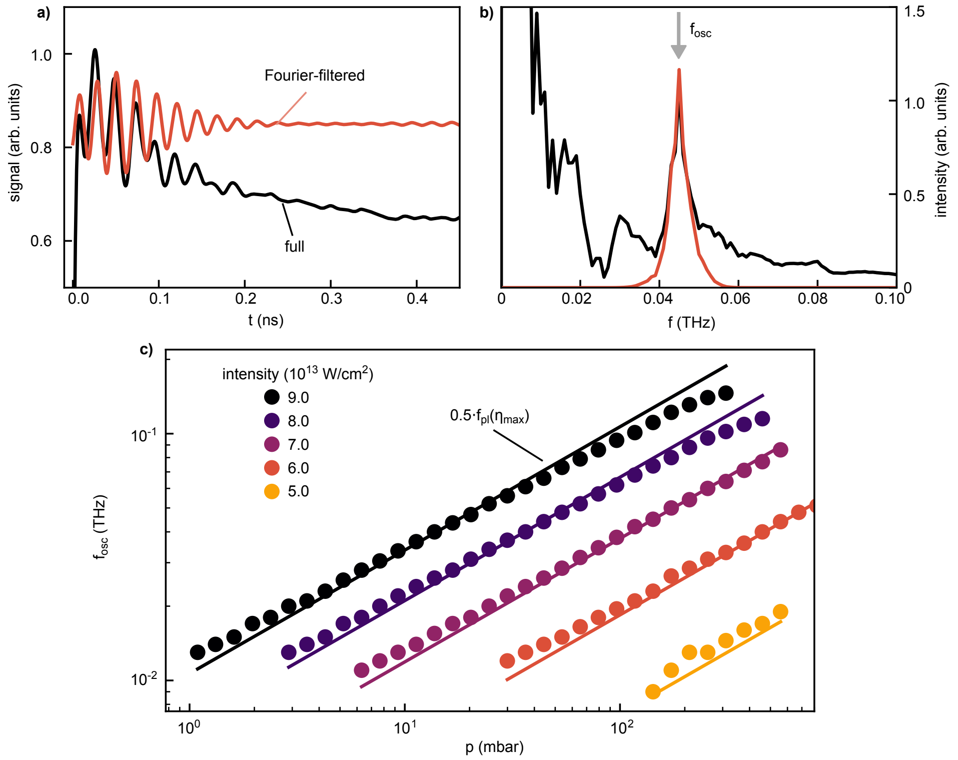

Supplementary Figure 1. Analysis of the plasma oscillations: a) close-up view on the induced charge signal (black line) for $p = 44$ mbar and $I = 8 \cdot 10^{13}\,\mathrm{W\,cm^{-2}}$ in nitrogen. b) Fourier-transformation of the induced charge signal. A distinct peak is observed at f_{osc}. Inverse Fourier-transformation filtered around the peak (red line) serves as a cross-check as shown in a). c) Retrieved oscillation frequencies for different pressures and driving laser intensities: a close scaling with the plasma frequency calculated from the peak charge density is observed such that the observed oscillations can be identified with plasma oscillations.

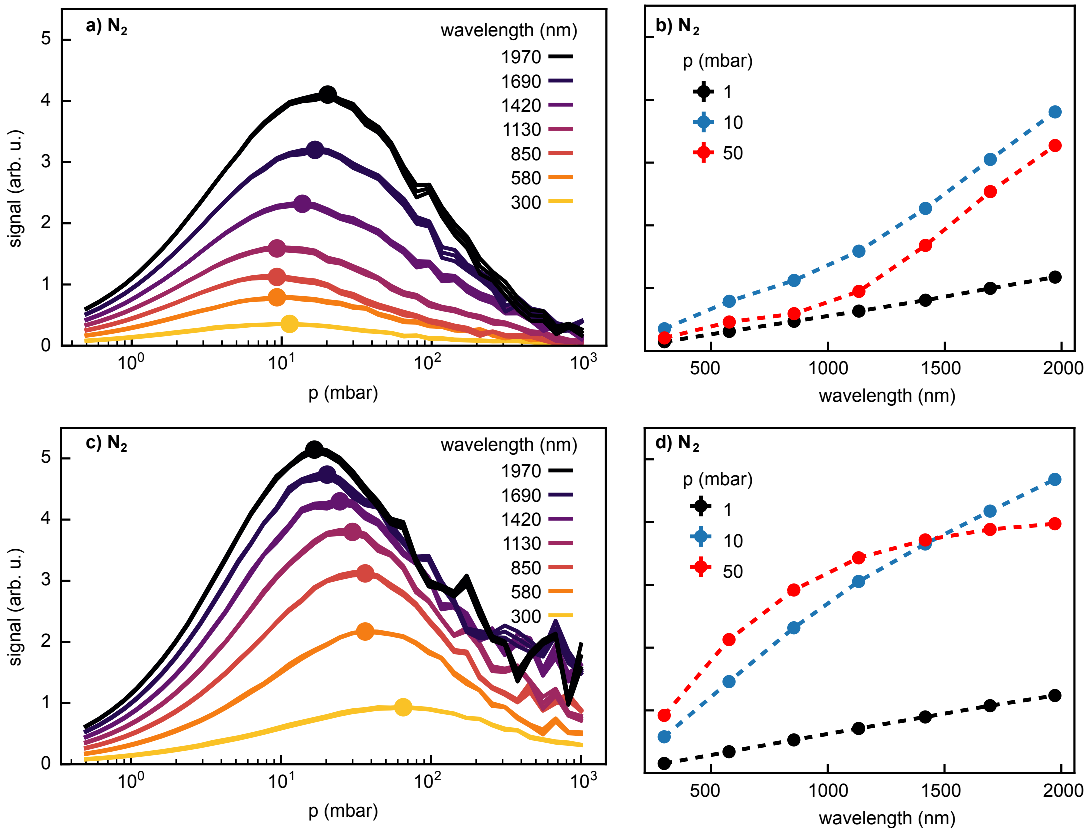

Supplementary Figure 2. Dependence of the signal strength on driving laser wavelengths. The peak intensity is $I = 8 \cdot 10^{13}\,\mathrm{W\,cm^{-2}}$ and the pulse duration is set to 1.75 cycles. a) pressure-dependence of the maximum induced charge signal for increasing wavelengths. The width of the lines represents the standard deviation of ten simulations with different random sampling of the initial electron distribution. b) the wavelength-dependence of the signal for selected pressures in nitrogen. At the lowest pressures, in the current regime, a linear dependence is observed which originates from the linear increase of the number of charges in nitrogen. c) and d) the same as a) and b) but for argon. The difference between argon and nitrogen in the simulations is purely due to the electron-neutral scattering.

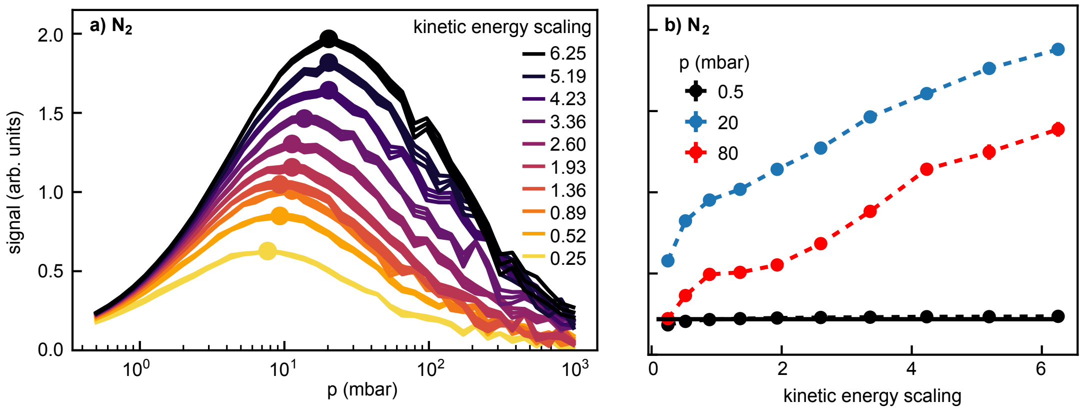

Supplementary Figure 3. Dependence of the induced charge signal on kinetic energy of the electrons: a) pressure dependence for different kinetic energies. The width of the lines represents the standard deviation of ten simulations with different random sampling of the initial electron distribution. b) kinetic energy scaling of the signal strength for different pressures. Only the kinetic energy of the electrons is scaled while all other simulation parameters are kept fixed ($I = 8 \cdot 10^{13}\,\mathrm{W\,cm^{-2}}$, $\omega_0 = 8\mu\mathrm{m}$, $\lambda = 750\,\mathrm{nm}$, $D = 85\mu\mathrm{m}$).
